# Account of an Expeditious Cure of a Fractured Scull

**Published:** 1784

**Authors:** 


### SECTION II.

### ESSAYS AND OBSERVATIONS.

I. *Account of an expeditious cure of a fractured ſcull. By Mr.* William Jones, *Surgeon at Birmingham; with remarks on the caſe by Mr.* Robert Mynors, *Surgeon of the ſame place. Communicated in a letter to Dr.* Simmons, *F. R. S.*

### CASE.

J. BURTON, a boy about five years old, on the 12th of September, 1783, received a wound on his head by a brick, which fell from the top of a chimney twelve yards high, and ſtruck him to the ground. He was immediately taken up ſenſeleſs, and conveyed to his father's houſe, which was only a few yards diſtant. I ſaw him in about half an hour after the accident, and found him in a comatoſe ſtate, vomiting frequently. His body and extremities were cold, and his pulſe ſmall. The wound bled freely, and was of a triangular ſhape. Upon introducing my finger into it, I felt a fracture of the cranium with depreſſion; this this determined me to apply the trephine, and I fent to Mr. Mynors, defiring the favour of his advice and affiftance in the operation. When he arrived, which was within an hour and a quarter after the accident, he recommended the wound of the fcalp to be dilated at each angle. Accordingly, I made two incifions acrofs the head, nearly over the angular junction of the occipital with the two parietal bones, and a third, which had its direction nearly in the courfe of the fagittal future, fo as to meet the other two at their junction over the angle above mentioned. Each of thefe incifions meafured about two inches and a half. The portions of the fcalp marked out by thefe incifions being carefully diffected from the pericranium, and raifed, the whole of the fracture, (which was fituated nearly in the middle, between the pofterior, fuperior and inferior angles of the left parietal bone, and almoft clofe to the lambdoidal future) prefented itfelf to our view. It had fomewhat of a femicircular form, and in circumference was more than equal to the fize of a half-crown piece, exclufive of feveral angular points made by the irregularity of the fracture. Mr. Mynors advifed an immediate application of the trephine to to that part of the ſound bone neareſt to the lambdoidal ſuture, in ſuch a manner, that the edge of the inſtrument might fall into the fracture; this appearing to be the moſt favourable part for the elevation of the depreſſed portion of bone, as well as the moſt depending. This was accordingly done after the previous uſe of the rugine; in applying which, however, care was taken to denude only ſo much of the bone as the crown of the trephine might be able to cover. On removing this circular piece of bone, I tried to elevate the depreſſed fractured portion, but without ſucceſs. A ſecond application of the trephine therefore became neceſſary; and this was made on the moſt prominent part of the bone, near the edge of the fracture, in the ſame manner as the firſt, from which it was diſtant about three quarters of an inch. At the particular requeſt of Mr. Mynors, I made no uſe of the rugine, as preparatory to this laſt application of the trephine.

After theſe perforations, a portion, as large as a halfpenny, of the depreſſed bone, which on our attempting to raiſe it with the elevator, was found to be looſe, was removed by means of a pair of forceps; the remaining part of the fractured and depreſſed piece was then elevated, and and every ſmall ſplinter and angular point of bone carefully removed. On the dura mater, thus expoſed to view, we found a ſmall quantity of extravaſated fluid and coagulated blood, which was very cautiouſly removed with a ſponge. Mr. Mynors now mentioned to me the uſe he intended to make of the flaps of ſcalp which had been ſo carefully raiſed from the pericranium, and preſerved intire, requeſting, at the ſame time, that he might dreſs the wound the firſt time after his own manner, which I readily agreed to. He began with moiſtening the inner ſurfaces of the flaps of the ſcalp, as well as the pericranium, and parts of the dura mater, which were now become dry by expoſure to the air, by touching them gently with a ſponge wrung out of warm water; after which he deſired me to apply my hands to the patient's head on each ſide of the wound, ſo as to preſs the flaps gently towards the centre, while he brought the edges of theſe flaps every where into eaſy and cloſe contact, and retained them in this ſituation by narrow and long ſlips of ſticking plaiſter, placed at ſmall diſtances from each other. The dreſſings conſiſted of a tow pledgit ſpread with wax and oil,

 and and a ſoft linen compreſs, and the whole was ſecured by a ſix-tailed bandage.

The patient was then carried to bed, and directed to be put on a low diet, and to take at ſtated times a mixture with antimonial wine and Thebaic tincture. He remained comatoſe during the firſt part of the night.

*Sept.* 13. I found that he had vomited once ſince the operation; but that in other reſpects he had paſſed a very good night. His heat was a little more than natural; his pulſe rather quick, and his ſkin moiſt. He made no complaint, and had had no more ſleep than might be attributed to the medicine he had taken.

*Sept.* 15. The pledgit whieh adhered to the ſcalp was removed only in part, and a freſh one applied over it.

*Sept.* 17. The former pledgits, as alſo the ſlips of plaſter, were this day removed entirely and new ones applied. The ſcalp was now found to be perfectly united every where to the pericranium, except a very ſmall portion of it, of about half the ſize of a little finger nail at the meeting of the angles of the flaps, where one of theſe angles had retracted for want of bony ſubſtance to ſupport it. In the courſe of the inciſions, a kindly but trifling digeſtion appeared, appeared, which diminiſhing daily, they healed in the ſpace of a fortnight. Granulations from the angles of the flaps of ſcalp, and from the ſmall portion of the dura mater, which was found uncovered after the firſt dreſſing, by degrees joined together, ſo that that membrane was completely covered in three weeks, and the whole wound was entirely healed in the ſpace of ſix weeks after the operation. It would have been cicatriſed ſooner, had not ſome ſcabs formed, which retarded the cure during the laſt fortnight. The ſmall portion of the dura mater found uncovered, as deſcribed above, and the inciſions of the ſcalp were continued during the whole of the cure to be dreſſed as at firſt, being only juſt covered with a pledgit thinly ſpread with wax and oil.

On the fifth day after the operation, the heat of the child's body and his pulſe appeared to be perfectly natural. He had no complaint after the firſt night, and when aſked how he did, always replied, that he was very well; ſo that no medical aſſiſtance was neceſſary, farther than occaſionally opening his bowels during the firſt week or ten days.

P. S. Yeſterday (June 2) I ſaw the boy, and had the pleaſure to find him in perfect health, and that nature was repairing the loſs of bony ſubſtance very faſt, the cicatrix appearing merely as a line, and producing no deformity, as the hair grows on the portions of ſcalp which were raiſed from the pericranium, as well as if no operation had ever taken place.

*Birmingham,*
June 3, 1784.

